# Photoreceptor Density–Dependent Kinetics of Geographic Atrophy Progression

**DOI:** 10.1016/j.xops.2026.101198

**Published:** 2026-04-17

**Authors:** Shinichiro Chujo, Alberto Quarta, Giulia Corradetti, Yu-Chien Chung, Hyunduck Kwak, Ceren Soylu, Rouzbeh Abbasgholizadeh, Mai Alhelaly, Raiyna Rattu, Jianfeng Huang, Swetha Velaga, Muneeswar Gupta Nittala, Akihito Uji, Srinivas R. Sadda

**Affiliations:** 1 Doheny Eye Institute, Pasadena, California; 2 Department of Ophthalmology David Geffen School of Medicine, University of California Los Angeles, Los Angeles, California; 3 Department of Ophthalmology, Mie University Graduate School of Medicine, Tsu City, Mie, Japan; 4 Department of Neurosciences, Imaging and Clinical Sciences, University “G. d'Annunzio” Chieti-Pescara, Chieti, Italy; 5 Department of Ophthalmology, Fu Jen Catholic University Hospital, Fu Jen Catholic University, New Taipei City, Taiwan; 6 Department of Ophthalmology, College of Medicine, The Catholic University of Korea, Seoul, Republic of Korea; 7 Ophthalmology Department, Tanta University, Egypt; 8 Chinese Academy of Medical Sciences, Institute of Geriatric Medicine, Beijing Hospital, National Center of Gerontology, Beijing, PR China; 9 Uji Eye Clinic, Yokkaichi City, Japan

**Keywords:** Geographic atrophy, Fundus autofluorescence, GA progression, Photoreceptor density

## Abstract

**Purpose:**

To investigate whether the spatial distribution of photoreceptor density governs the rate and directionality of geographic atrophy (GA) progression and to propose a photoreceptor density–based metric for biologically informed assessment of GA progression.

**Design:**

A retrospective longitudinal analysis of untreated fellow eyes from a multicenter, randomized, sham-controlled clinical trial (MAHALO).

**Participants:**

A total of 103 untreated fellow eyes from 143 eyes enrolled in the MAHALO study, all with GA secondary to age-related macular degeneration.

**Methods:**

Fundus autofluorescence images obtained at baseline and 18 months were analyzed. Geographic atrophy lesion area and boundary progression distance (“front”) were quantified using rigidly registered images. The retina was divided into concentric eccentricity rings centered on the fovea. Theoretical cone and rod density models derived from histologic data were applied to generate photoreceptor density maps. Photoreceptor loss within GA lesions was estimated, and a photoreceptor-adjusted front was calculated by weighting boundary progression by local photoreceptor density. Directional progression was further evaluated using clock-hour–based analysis and a cosine-based directional metric (cosθ).

**Main Outcome Measures:**

Geographic atrophy area enlargement, front progression distance, photoreceptor-adjusted front, ring-wise photoreceptor loss, and directional variability of lesion expansion.

**Results:**

Geographic atrophy enlargement differed significantly across eccentricity rings (*P* < 0.001), demonstrating relatively slower expansion in regions with higher modeled photoreceptor density and greater expansion in lower-density regions. Total photoreceptor loss showed a moderate positive correlation with GA area enlargement (r = 0.48; *P* = 0.001). Directional variability of progression was significantly reduced when using the photoreceptor-adjusted front compared with the geometric front (coefficient of variation: 0.769 vs. 1.145; *P* < 0.01). In extrafoveal lesions, conventional front measurements demonstrated more rapid peripheral versus foveal progression, whereas this directional asymmetry was attenuated after photoreceptor density adjustment.

**Conclusions:**

Geographic atrophy progression may be partly explained by the spatial distribution of photoreceptor density, among other contributing biological factors. Apparent directional and regional differences in GA expansion may reflect underlying gradients in photoreceptor density in addition to geometric and other biological influences. Photoreceptor density–based metrics provide a biologically informed framework for interpreting GA progression and may serve as complementary measures for natural history studies and clinical trials.

**Financial Disclosure(s):**

Proprietary or commercial disclosure may be found in the Footnotes and Disclosures at the end of this article.

Geographic atrophy (GA) is one of the leading causes of visual impairment worldwide.^1^^,^^2^ More than 5 million people are affected globally, and its prevalence is expected to increase further with the ongoing aging of the population.^3^ Once GA develops, the lesions inevitably enlarge and often result in severe central vision loss.^4^

In recent years, the approval of complement inhibitors and advances in gene therapy have made it increasingly realistic to target the attenuation of GA progression as a therapeutic goal.^5^, ^6^, ^7^, ^8^, ^9^, ^10^ Several clinical trials are currently underway; however, most of them use “enlargement rate of lesion area” as the primary outcome measure, which does not necessarily reflect changes in visual function accurately.^11^ It has also been reported that extrafoveal lesions progress faster than foveal lesions and that lesions tend to expand more rapidly toward the periphery than toward the fovea.^12^^,^^13^ However, the biological mechanisms underlying such patterns of progression have not been fully investigated. To appropriately evaluate the efficacy of future therapeutic interventions, a deeper understanding of the pathophysiological basis of GA progression is essential. To characterize GA progression, several structural biomarkers have been proposed. These include the distance from the lesion margin to the foveal center and OCT-based markers such as photoreceptor and ellipsoid zone loss, external limiting membrane disruption, outer nuclear layer thinning, retinal pigment epithelium (RPE) attenuation, and choroidal hypertransmission.^14^, ^15^, ^16^

These biomarkers have provided important prognostic information regarding visual outcomes and lesion expansion risk. However, they primarily assess local or partial structural alterations and do not explicitly incorporate broader biologic and anatomic constraints that may influence the directionality and asymmetry of GA expansion. Consequently, the underlying biologic basis of spatial progression patterns remains incompletely understood. Such a biologically constrained framework may provide complementary information for patient stratification and endpoint interpretation in future interventional trials.

In general, GA progression has been explained because of RPE damage leading to secondary cell death in neighboring cells.^17^^,^^18^ In addition to this mechanism, we focused on the possibility that the distribution density of photoreceptors may influence the rate and directionality of GA progression. Curcio et al have reported in detail the density distribution of cones and rods in the human retina,^19^, ^20^, ^21^ and based on this biological model, we hypothesized that the local photoreceptor density may determine the susceptibility of retinal tissue to GA expansion. The primary aim of this study was to present a conceptual and mechanistic framework to examine the extent to which the rate and directionality of GA expansion can be explained by photoreceptor density as a biologic and anatomic constraint. Specifically, we tested the hypotheses that lesion enlargement, as a function of distance, is relatively attenuated in regions of high photoreceptor density and that the spatial distribution of photoreceptors contributes to asymmetry in the speed and direction of GA progression.

## Methods

### Study Design

The MAHALO study (NCT01229215) was a phase Ib/II multicenter, randomized, single-masked, sham-controlled trial evaluating the safety, tolerability, and activity of lampalizumab in patients with GA secondary to age-related macular degeneration. Eligible patients had bilateral GA without any history or evidence of macular neovascularization. The study adhered to the tenets of the Declaration of Helsinki and was approved by the Institutional Review Board of the University of California, Los Angeles (approval number: IRB-15-0083). For this analysis, we included 103 untreated fellow eyes that received neither lampalizumab nor sham injections and had fundus autofluorescence (FAF) images available at all scheduled visits. These eyes provided longitudinal imaging data suitable for evaluating the natural course of GA progression.

### Image Acquisition and Processing

Fundus autofluorescence images were acquired using a 30° field centered on the fovea with the Spectralis HRA + OCT system (Heidelberg Engineering). Images obtained at 2 time points—baseline (0 months) and 18 months—were included for analysis. At each time point, GA lesions were semi-automatically delineated by a certified grader (M.G.) at the Doheny Image Reading and Research Lab using the Spectralis Region Finder software and were rereviewed by a second certified grader (S.C.) to correct any segmentation errors and ensure precise delineation. All segmentation mask images of the GA lesion were managed and processed using Image J software (National Institutes of Health). The foveal center was manually identified by referencing multiple imaging modalities, including color fundus photography, FAF, and OCT, to ensure consistency across all time points. To ensure accurate measurement of boundary displacement/advancement, all follow-up FAF images were rigidly registered to the baseline image using the enhanced correlation coefficient (ECC) algorithm. The ECC algorithm is an iterative optimization method that maximizes the correlation coefficient between images, making it well suited for intensity-based registration and enabling more accurate image alignment.^22^ Registration was performed using the foveal center and overall lesion morphology. To demonstrate the accuracy of this method, we compared lesion mask images generated from FAF images before and after ECC-based registration and reported the ECC correlation coefficients and pixel-level displacement values in Figure S1 and Table S1 (available at www.ophthalmologyscience.org).

### Assessment of GA Lesion Area and Front Progression

#### Lesion Area

The GA lesion area was measured at baseline and follow-up, and the area enlargement was calculated as:

$$\text{Area}\;\text{progression}\left({mm}^{2}\right)={\text{Area}}_{\text{follow}-\text{up}}–{\text{Area}}_{\text{baseline}}$$

#### Front: A Distance-Based Metric of GA Lesion Progression

**Figure S2:**
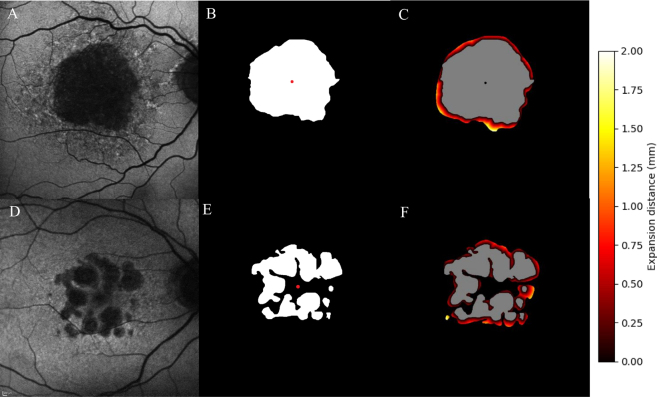
Progression heatmap used for geographic atrophy front calculation. (A) Original fundus autofluorescence (FAF) image. (B) Binary mask image generated by semi-automatically delineating GA lesion margins on the FAF image using the Spectralis Region Finder software. (C) Heatmap image visualizing lesion margin expansion, created by applying a Euclidean distance map to the baseline and follow-up mask images. (D–F) In eyes with multiple GA lesions, each lesion was independently segmented and divided into 12 clock-hour sectors. Sectoral distances were then integrated to assess the overall directional expansion pattern.

To quantitatively evaluate local GA lesion progression, we used our previously reported method, which divides GA lesions into 12 clock-hour sectors and calculates the progression distance in each direction.^23^ A Euclidean distance map was first generated from the baseline mask, upon which the follow-up mask was overlaid to extract peripheral distance profiles. Sectoral expansion distances were then measured across 12 clock-hour directions, and the average value was calculated to visualize the GA progression as a heatmap (Fig S2, available at www.ophthalmologyscience.org). The average of these sector-wise distances was defined as the GA front. In eyes with multiple GA lesions, each lesion was segmented separately, and its boundary was divided into 12 clock-hour sectors to measure the front distance. The results from all lesions were averaged to quantify the overall pattern of atrophy expansion (Fig S2). This value is referred to as the “front” throughout the remainder of the manuscript.

### Photoreceptor Density–Based Metric

#### Photoreceptor Density Distribution

To estimate photoreceptor loss within GA lesions, theoretical cone and rod distributions were used to compute the number of photoreceptors lost within the GA area, based on previously established histologic models. Approximate theoretical values for retinal photoreceptor densities were derived based on previous publications by Curcio et al and are listed in Table S2 (available at www.ophthalmologyscience.org).^19^, ^20^, ^21^

Cone density reaches its maximum within the 0 to 0.5 mm region from the foveal center and gradually declines beyond approximately 1 mm. In contrast, rod density is low directly beneath the fovea but peaks around 2 to 3 mm from the center. When these distributional characteristics are integrated, the total photoreceptor density presents a bimodal pattern, with 2 distinct peaks observed at 0 to 0.5 mm and 2 to 3 mm from the foveal center. The photoreceptor density used in this study was not measured longitudinally in individual eyes. Instead, a position-dependent theoretical photoreceptor density map derived from histologic data was applied. This density distribution was treated as a static anatomic reference reflecting inherent retinal structure. The photoreceptor-adjusted front was therefore calculated by weighting front displacement according to the local photoreceptor density at each retinal location, representing the anatomic substrate through which the GA boundary advances.

#### Photoreceptor Density Map

To further examine spatial variation, the retina was divided into 5 concentric eccentricity rings: 0 to 0.5 mm, 0.5 to 1 mm, 1 to 2 mm, 2 to 3 mm, and 3 to 5 mm from the foveal center. Cone and rod loss were computed independently within each ring by summing photoreceptor density values at each GA pixel within the corresponding ring.

**Figure 1:**
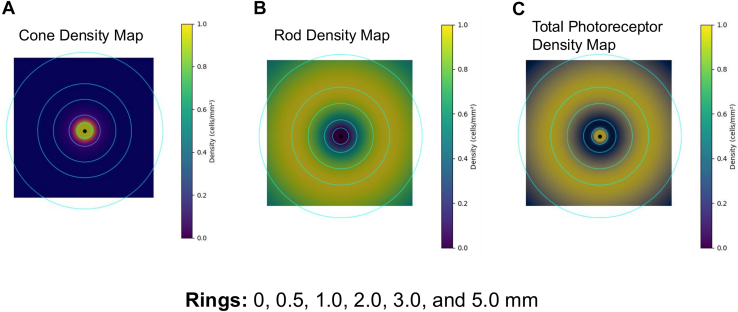
Photoreceptor density distribution maps based on cone, rod, and total photoreceptors. **A,** Cone density map. **B,** Rod density map. **C,** Total photoreceptor map. In each map, regions of higher density are shown in yellow, corresponding to areas of increased photoreceptor density as defined by the photoreceptor density distribution. The retina was divided into concentric rings centered on the fovea: 0 to 0.5 mm, 0.5 to 1 mm, 1 to 2 mm, 2 to 3 mm, and 3 to 5 mm.

**Figure 2:**
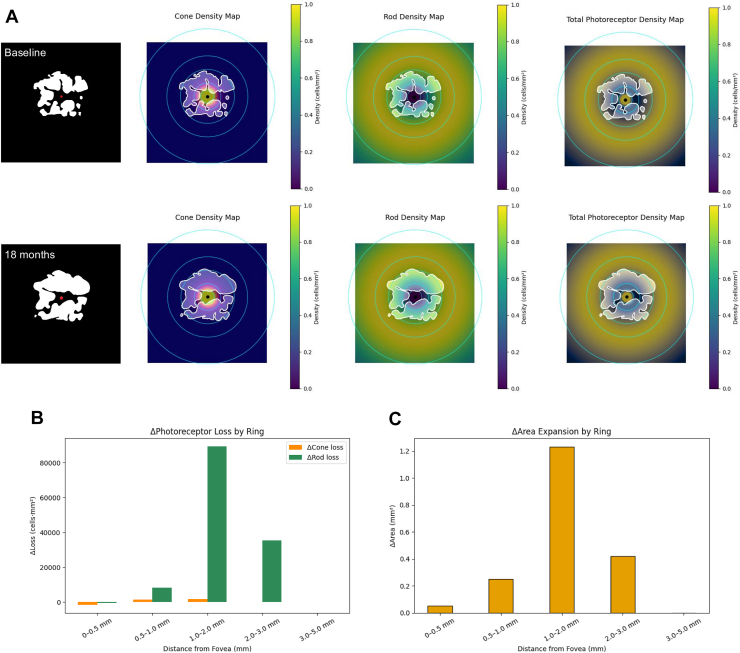
Photoreceptor density distribution maps with GA lesions. **A,** By overlaying these photoreceptor density maps with the GA lesion masks. The red dot denotes the foveal center. It is possible to assess which photoreceptor density regions—corresponding to specific concentric rings—the lesions preferentially expand into. **B,** The increase in cell loss (Δphotoreceptor loss) was calculated for each concentric ring. **C,** The increase in area (ΔArea) was calculated for each concentric ring. GA = geographic atrophy.

Based on these data, we generated a cone density map, rod density map, and total photoreceptor map (Fig 1). In each map, regions of high density are highlighted in yellow, corresponding well to the known photoreceptor density distribution. By overlaying these density maps onto the GA lesion masks, we were able to evaluate which photoreceptor density zones—specifically, which concentric rings—the lesion expansion corresponded to (Fig 2). This approach enabled analysis of GA progression not merely in terms of distance or area, but in relation to the local photoreceptor density.

#### Quantification of Cone Loss

Photoreceptor loss was quantified as the sum of the products of cone density and pixel area across all pixels within the GA lesion. Cone density at each pixel was defined using an exponential model based on the distance r (mm) from the foveal center, as follows:

$$\text{cone}\_\text{density}\left(r\right)=200,000\cdot e^{–r/0.3}$$

Here, r represents the distance from the foveal center (mm), and 200 000 denotes the theoretical maximum cone density at the foveal center (cells/mm^2^). This model assumes an exponential decay in which the density decreases to approximately 37% (1/e) with every 0.3 mm increase in eccentricity. For example, when r = 1 mm, the density drops to approximately 3.6% of its peak value.

The exponential decay constant τc = 0.3 mm was defined based on histologic analysis of the human retina by Curcio et al, representing the spatial scale at which cone density declines to 1/e with increasing distance from the foveal center.^19^, ^20^, ^21^ Assuming that all cones are completely lost within the GA lesion, the theoretical number of cones lost (cone loss) was calculated by summing the product of cone density and pixel area for each pixel within the GA lesion, as follows:

$$\text{Cone}\;\text{loss}\;\left(\text{cells}\right)=\sum \left({\text{cone}}_{\text{density}\left(r\right)\left[\text{cells}/{\text{mm}}^{2}\right]}\times \text{picel}\;\text{area}\;\left[{\text{mm}}^{2}\right]\right)$$

#### Quantification of Rod Photoreceptor Loss

Rod photoreceptor loss was quantified as the sum of the products of rod density and pixel area across all pixels within the GA lesion. Rod density at each pixel was defined using a gamma-like distribution model based on the distance from the foveal center in millimeters, as follows:

$$\text{rod}\_\text{density}\left(r\right)=90,000\cdot \left(\frac{r}{3.0}\right)\cdot e^{1–r/3.0}$$

Here, r represents the distance from the foveal center in millimeters, and 90 000 is a scaling constant representing the approximate peak rod density (cells/mm^2^). This model reflects known physiological characteristics whereby rod density is zero directly beneath the fovea (at r = 0), reaches a maximum at approximately 3 mm from the center, and then declines exponentially.

These features are supported by histologic studies of the human retina conducted by Curcio et al, which demonstrated that rod density peaks at an eccentricity of approximately 3 mm.^19^, ^20^, ^21^ This distribution follows a gamma-like curve, making it well-suited to replicate both the smooth initial rise and subsequent exponential decay of rod density. Based on this function, the theoretical rod loss was calculated by summing the product of rod density and pixel area for all pixels within the GA lesion, as follows:

$$\text{Rod}\;\text{loss}\;\left(\text{cells}\right)=\sum \left(\text{rod}\_\text{density}\left(r\right)\;\left[{\text{cells}/\text{mm}}^{2}\right]\times \text{pixel}\;\text{area}\;\left[{\text{mm}}^{2}\right]\right)$$

#### Quantification of Total Photoreceptor Density

Total photoreceptor density was defined as the sum of cone and rod densities and was calculated for each pixel based on its distance from the foveal center. The density distribution models for cones and rods were as described earlier, and total photoreceptor density was obtained by summing these 2 components. The theoretical total photoreceptor loss was calculated by summing the product of total photoreceptor density and pixel area across all pixels within the GA lesion, and is expressed by the following equation:

$$\text{Total}\;\text{loss}\;\left(\text{cells}\right)=\sum \left(\text{total}\_\text{photoreceptor}\_\text{density}\left(r\right)\;\left[{\text{cells}/\text{mm}}^{2}\right]\times \text{pixel}\;\text{area}\;\left[{\text{mm}}^{2}\right]\right)$$

#### Photoreceptor-Adjusted Front: A Density-Based Metric of GA Expansion

The photoreceptor-adjusted front is a metric calculated by multiplying the previously defined GA lesion front (in mm) by the local total photoreceptor density at the baseline lesion border position corresponding to each direction of expansion. This index approximates the number of photoreceptors lost per unit distance of lesion expansion and can be interpreted as a rate-like metric that reflects the extent of photoreceptor degeneration.

The calculation is performed by multiplying the front value (mm) by the total photoreceptor density (cells/mm^2^) at the corresponding baseline location, resulting in the number of photoreceptors affected per millimeter (cells/mm), as expressed by the following equation:

$$\text{Photoreceptor}-\text{Adjusted}\;\text{Front}\;\left[\text{cells}/\text{mm}\right]=\text{Front}\;\left[\text{mm}\right]\times \text{Total}\;\text{Photoreceptor}\;\text{Density}\;\left[{\text{cells}/\text{mm}}^{2}\right]$$

### Ring-Wise Evaluation of the Relationship between GA Area Expansion and Photoreceptor Density

Based on the central hypothesis of this study, we conducted analyses using the previously defined concentric ring regions to reflect the photoreceptor density gradient relative to the distance from the foveal center. For all 103 eyes, GA lesion pixels contained within each ring region were extracted, and cone loss, rod loss, and total photoreceptor loss were calculated for each ring based on the theoretical density models.

Photoreceptor loss within each ring was computed by summing the theoretical photoreceptor density values corresponding to the GA lesion pixels within that ring. In parallel, the increase in lesion area (ΔArea) within each ring was measured, and its association with photoreceptor loss metrics (cone loss, rod loss, and total photoreceptor loss) was evaluated. To further examine how the relationship between photoreceptor loss and GA lesion expansion varies according to photoreceptor density levels, scatter plots were generated for each ring region, with photoreceptor loss metrics on the x-axis and the corresponding ΔArea on the y-axis. These plots enabled a quantitative assessment of the relationship between local photoreceptor loss and lesion area enlargement. These analyses were conducted to evaluate the extent to which GA lesion area enlargement can be explained by photoreceptor loss derived from photoreceptor density.

In addition, because ΔArea within each ring may be affected by geometric bias arising from differences in ring surface area and baseline lesion morphology, we additionally performed ring-wise analyses using front, a metric less influenced by such geometric factors, to further strengthen the robustness of our findings.

### Assessment of GA Lesion Front Expansion and Directionality in Relation to Photoreceptor Density

#### Overall Trend between GA Progression Direction and Photoreceptor Density

To test the hypothesis that the spatial distribution of photoreceptor density contributes to the directionality and asymmetry of GA progression, we first performed a global assessment of the relationship between lesion expansion direction and local photoreceptor density. Specifically, the lesion margin was divided into 12 clock-hour sectors for all 103 eyes in the cohort, and the front (i.e., lesion expansion distance perpendicular to the lesion border) was calculated for each direction.

In addition, using the photoreceptor-adjusted front—which incorporates the theoretical local photoreceptor density in the same direction—we quantified the directional variability of lesion progression by calculating the coefficient of variation (CV) of the expansion distance across the 12 clock-hour sectors. The CVs of the front and photoreceptor-adjusted front were then compared to assess the extent of directional variation in each metric.

#### Detailed Directional Analysis Using the Cosθ Metric in Extrafoveal Lesions

In the preceding global analysis, directional effects could potentially be averaged out due to intercase variability in lesion expansion direction or lesion location (e.g., fovea involved or not). Thus, to more precisely assess the directionality of lesion progression, a subgroup analysis was performed, limited to cases with extra-foveal GA lesions that did not involve the foveal center at baseline (n = 38).

For these cases, the front and photoreceptor-adjusted front values were individually calculated for each lesion in both the foveal and peripheral directions, and differences between directions were assessed.

Furthermore, for each lesion edge pixel, the direction of lesion expansion was defined using the cosine of the angle (cosθ) between the pixel's expansion vector and the vector toward the foveal center. Cosθ was used as a continuous directional index, where a value of +1 indicates progression toward the fovea and –1 indicates progression away from the fovea. This approach was adapted from a directional analysis metric previously used in studies of brain tumor expansion.^24^

Previous studies have primarily evaluated GA progression using a binary classification of directions—either toward the fovea or toward the periphery.^12^ In the present study, building upon this framework, each lesion edge pixel was classified using the cosθ value as follows:

• Foveal direction: acute angle toward the fovea (angle 0°–90°, cosθ > 0).
• Peripheral direction: obtuse angle away from the fovea (angle 90°–180°, cosθ < 0).
• Neutral: pixels with cosθ = 0 were excluded from analysis.

A conceptual overview of lesion edge expansion directions based on cosθ is shown in Figure S3 (available at www.ophthalmologyscience.org). A summary of the correspondence between cosθ values and directional classification is presented in Figure S3 and Supplementary Table S3 (available at www.ophthalmologyscience.org).

**Figure 3:**
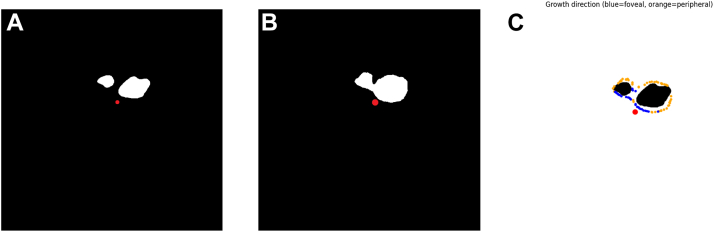
Cosine-based classification of GA lesion expansion into macular and peripheral directions. **A,** GA lesion masks at baseline. The red dot denotes the foveal center. **B,** GA lesion masks at 18 months. The red dot denotes the foveal center. **C,** The baseline lesion is shown in black. The red dot denotes the foveal center. The margins of the GA lesion at 18 months are highlighted in blue and yellow, where blue indicates lesion expansion toward the macular direction and yellow indicates lesion expansion toward the peripheral direction. GA = geographic atrophy.

Using this approach, lesion edge pixels were categorized into foveal and peripheral directions, allowing for direction-specific quantitative analyses (Fig 3). In addition, for each case, we generated paired difference plots comparing the peripheral and foveal values of front and photoreceptor-adjusted front.

#### Supplementary Analysis of GA Expansion Direction Using Cosθ as a Continuous Variable

In the binary classification of lesion expansion direction (foveal vs. peripheral), the use of a threshold-based categorization may not fully capture the continuous nature of directional variation. Therefore, a supplementary analysis was performed in which cosθ (cosine value) was treated as a continuous variable. For each of the 38 previously selected eyes, cosθ was calculated for all pixels along the lesion edge, and the corresponding values of front and photoreceptor-adjusted front were obtained. Because these 2 metrics differ in numerical scale and units, normalization was performed within each eye by dividing by the respective median value.

The range of cosθ (–1 to +1) was divided into equal-width bins, and the mean normalized front value within each bin was calculated. These average values were then plotted against cosθ to visualize the overall trend between lesion expansion direction and progression magnitude across all pixels. This analysis was not intended for binary directional comparison, but rather to continuously evaluate the directional dependence of front and photoreceptor-adjusted front, and to compare how this dependency differs between the 2 metrics.

### Statistical Analysis

To compare GA lesion area enlargement across concentric ring regions (0–0.5 mm, 0.5–1.0 mm, 1.0–2.0 mm, 2.0–3.0 mm, and 3.0–5.0 mm), a linear mixed-effects model was used, with ring (reference: 0–0.5 mm) included as a fixed effect and eye included as a random effect to account for within-eye correlation. The overall effect of ring was assessed using a likelihood ratio test. For correlation analyses between total photoreceptor loss and GA lesion area enlargement in the scatter plots, Spearman rank correlation coefficient was used.

To compare to CV of front progression distances with and without photoreceptor-adjusted weighting, a paired *t* test was employed. For comparison of front values in the foveal and peripheral directions, the mean and standard deviation for each direction across all cases were calculated. Given the nonnormal distribution of the data, the Wilcoxon signed-rank test was used for paired comparisons. All statistical analyses were performed using Python (version 3.10), and *P* value of <0.05 was considered statistically significant.

## Results

### Ring-Wise Results Linking GA Expansion and Photoreceptor Density

**Figure 4:**
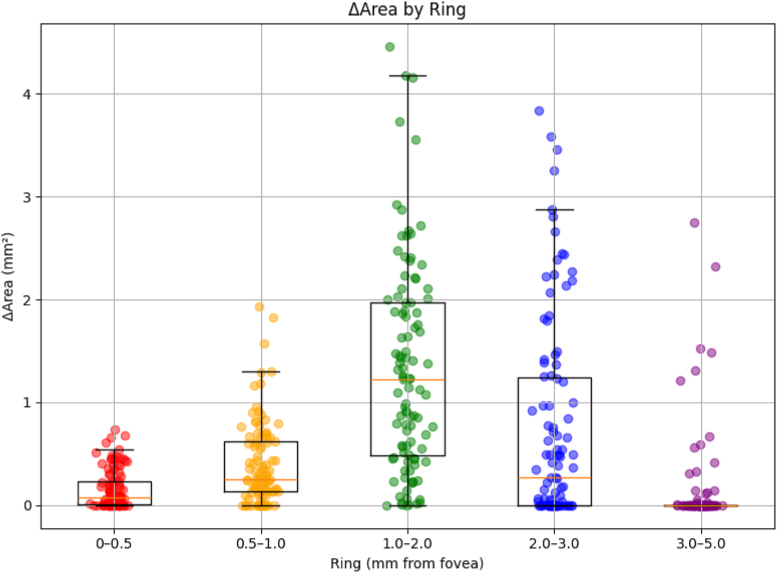
Ring-wise results linking GA area expansion and photoreceptor density. Geographic atrophy lesion progression was assessed using concentric fovea-centered rings (0–0.5 to 3–5 mm). ΔArea differed across rings, with minimal expansion centrally, a broad peak spanning 1 to 3 mm, and a decrease in the outermost ring. This nonlinear pattern spatially corresponded to the bimodal distribution of total photoreceptor density predicted by the theoretical model. ΔArea = increase in lesion area; GA = geographic atrophy.

To investigate the relationship between GA lesion progression and local photoreceptor density, GA enlargement was quantified within concentric ring regions centered on the fovea (0–0.5 mm, 0.5–1 mm, 1–2 mm, 2–3 mm, and 3–5 mm). In the mixed-effects model with the 0 to 0.5 mm ring as the reference, ΔArea differed significantly across rings (overall likelihood ratio test *P* < 0.001). Compared with the central ring, the 0.5 to 1.0 mm (β = +0.243 mm^2^, *P* < 0.01), 1 to 2 mm (β = +1.161 mm^2^, *P* < 0.001), and 2 to 3 mm rings (β = +0.589 mm^2^, *P* < 0.001) demonstrated significantly greater area enlargement, whereas the outermost 3 to 5 mm ring did not differ significantly from the reference (β = –0.021 mm^2^, *P* = 0.818) (Fig 4, Table 1). A similar ring-dependent pattern was observed for lesion front displacement (overall likelihood ratio test *P* < 0.001).

**Table 1:** Ring-Wise Mixed-Effects Model Results for Area

Ring (mm)	β (mm^2^)	95% CI	*P* Value
0.5–1.0	+0.243	0.065–0.421	<0.01
1.0–2.0	+1.161	0.983–1.339	<0.001
2.0–3.0	+0.589	0.411–0.767	<0.001
3.0–5.0	–0.021	–0.199 to 0.157	0.818
Overall ring effect (LRT)			<0.001

CI = confidence interval; LRT = likelihood ratio test.

Values are presented as regression coefficients (β) with 95% CIs. The reference ring was 0–0.5 mm. The overall ring effect was assessed using an LRT. Ring = Concentric annular zones centered on the fovea (0–0.5 mm, 0.5–1 mm, 1–2 mm, 2–3 mm, and 3–5 mm), defined based on photoreceptor density distribution.

**Figure 5:**
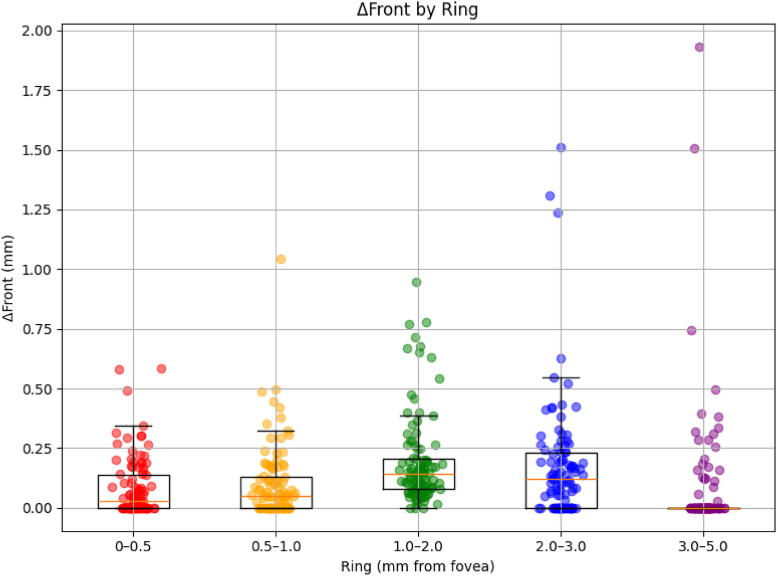
Ring-wise results linking GA front and photoreceptor density. Geographic atrophy lesion progression was assessed using concentric fovea-centered rings (0–0.5 to 3–5 mm). Front differed across rings, with minimal expansion centrally, a broad peak spanning 1 to 3 mm, and a decrease in the outermost ring. This nonlinear pattern spatially corresponded to the bimodal distribution of total photoreceptor density predicted by the theoretical model. GA = geographic atrophy.

Front was significantly greater in the 1 to 2 mm (β = +0.112 mm, *P* < 0.001) and 2 to 3 mm rings (β = +0.090 mm, *P* < 0.001), whereas no significant differences were detected in the 0.5 to 1.0 mm (β = +0.016 mm, *P* = 0.476) or 3 to 5 mm rings (β = +0.002 mm, *P* = 0.935) compared with the reference (Fig 5, Table 2). Overall, both ΔArea and front demonstrated a relative peak across the parafoveal region (1–3 mm) with attenuation toward the outermost ring. This spatial pattern was broadly consistent with the theoretical bimodal distribution of total photoreceptor density derived from histologic models.

**Table 2:** Ring-Wise Mixed-Effects Model Results for Front

Ring (mm)	β (mm)	95% CI	*P* Value
0.5–1.0	+0.016	–0.028 to 0.061	0.476
1.0–2.0	+0.112	0.067–0.157	<0.001
2.0–3.0	+0.090	0.045–0.134	<0.001
3.0–5.0	+0.002	–0.043 to 0.046	0.935
Overall ring effect (LRT)			<0.001

CI = confidence interval; LRT = likelihood ratio test.

Values are presented as regression coefficients (β) with 95% CIs. The reference ring was 0–0.5 mm. The overall ring effect was assessed using an LRT. Ring = Concentric annular zones centered on the fovea (0–0.5 mm, 0.5–1 mm, 1–2 mm, 2–3 mm, and 3–5 mm), defined based on photoreceptor density distribution.

**Figure 6:**
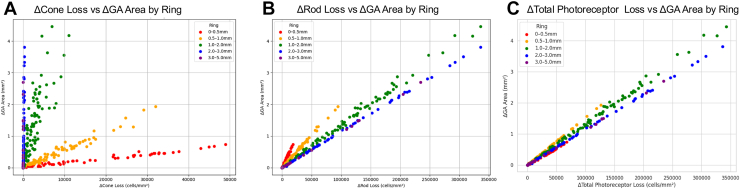
Correlation plot of photoreceptor loss and GA area expansion. **A,** For cones, cell loss was greatest in the central high-density region (0–0.5 mm), whereas GA area expansion (ΔArea) remained relatively small; in more peripheral rings, cone loss was smaller but ΔArea tended to be larger. **B,** For rods, both cell loss and ΔArea increased with eccentricity. **C,** Combined cone and rod loss (total photoreceptors) showed a moderate positive correlation with GA area expansion (r = 0.48; *P* = 0.001). ΔArea = increase in lesion area; GA = geographic atrophy.

With respect to cone photoreceptors, the highest cone loss was observed in the central region (0–0.5 mm, shown in red), where cone density is maximal. Despite this high level of photoreceptor loss, the corresponding ΔArea remained relatively small. In contrast, in more peripheral rings, cone loss was lower, but ΔArea tended to be greater. For rod photoreceptors, the spatial increase in rod loss toward the periphery reflected the anatomical trend of increasing rod density with eccentricity. Importantly, greater rod loss was generally accompanied by an increased ΔArea in the same regions. Furthermore, a moderate positive correlation was observed between total photoreceptor loss (cone + rod) and ΔArea across rings (r = 0.48, *P* = 0.001) (Fig 6). Thus, the apparent reduction in central GA area expansion relative to peripheral regions may reflect underlying differences in photoreceptor density.

### Overall Directional Results in Relation to Photoreceptor Density

**Figure 7:**
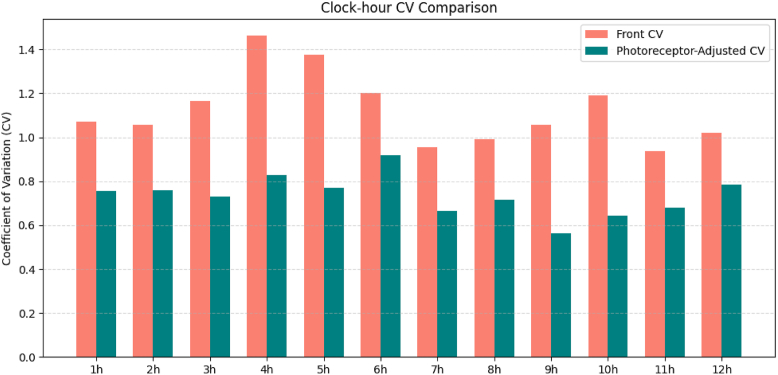
Clock-hour–wise variability of front and photoreceptor-adjusted front in the overall cohort. The coefficient of variation (CV) for each clock-hour direction (1–12 o'clock) is shown as bar graphs. Front exhibited higher CV values across multiple directions, whereas photoreceptor-adjusted front demonstrated consistently lower CV values across all directions.

Quantitative evaluation of directional variability in lesion progression revealed that the CV across directions was significantly lower for the photoreceptor-adjusted front compared to the unadjusted front. Specifically, the mean CV for the front was 1.145 (standard deviation: 0.212), whereas the mean CV for the photoreceptor-adjusted front was 0.769 (standard deviation: 0.255), showing a significant reduction (*P* < 0.01) (Table 3). Furthermore, when the CV was visualized for each of the 12 clock-hour sectors using bar graphs, higher CV values were observed across many directions for the front, while consistently lower CV values were noted in all directions for the photoreceptor-adjusted front (Fig 7). These findings indicate that applying photoreceptor density-based correction reduces directional variability in GA lesion edge progression, suggesting an overall smoothing effect on directional asymmetry.

**Table 3:** Overall Cohort Variability of Front and Photoreceptor-Adjusted Front

Metric	Mean	SD	CV	CV Comparison (*P* Value)
Raw front (mm)	0.185	0.212	1.145	
Photoreceptor-adjusted front (cells/mm)	332 106	255 321	0.769	<0.01

CV = coefficient of variation; SD = standard deviation.

The *P* value represents the paired comparison of CVs between the 2 metrics using a paired *t* test.

### Directional Results in Extrafoveal Lesions Using the Cosθ Metric

**Figure 8:**
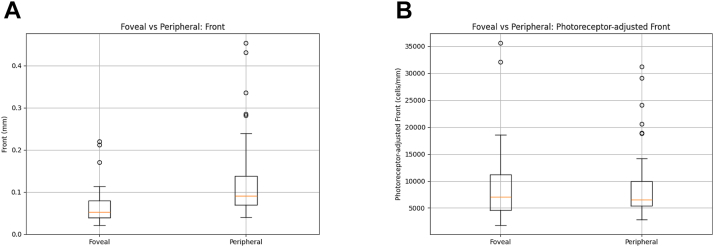
Box-and-Whisker comparison of front and photoreceptor-adjusted front in macular and peripheral directions. **A,** For the conventional front metric, expansion distance was greater in the peripheral than in the macular direction (*P* < 0.01), consistent with preferential peripheral GA progression. **B,** In contrast, photoreceptor-adjusted front showed no significant directional difference (*P* = 0.537). GA = geographic atrophy.

Using cosθ, calculated based on the vector relationship between each lesion edge pixel and the foveal center, lesion progression was classified into foveal and peripheral directions. Comparison of front and the photoreceptor-adjusted front in these directional categories revealed that, for the unadjusted front, the peripheral direction showed significantly greater expansion than the foveal direction (*P* < 0.01), consistent with previous reports that GA lesions tend to expand more toward the periphery than toward the fovea (Table 4, Fig 8).

**Table 4:** Comparison of Front and Photoreceptor-Adjusted Front between Foveal and Peripheral Directions

Metric	Region	Mean ± SD	Wilcoxon *P* Value
Front (mm)	Foveal	0.069 ± 0.046	<0.01
	Peripheral	0.129 ± 0.102	
Photoreceptor-adjusted front (cells/mm)	Foveal	9171 ± 7467	0.537
	Peripheral	9441 ± 7058	

SD = standard deviation.

The *P* values indicate the statistical comparison between foveal and peripheral directions for both the front and photoreceptor-adjusted front.

**Figure 9:**
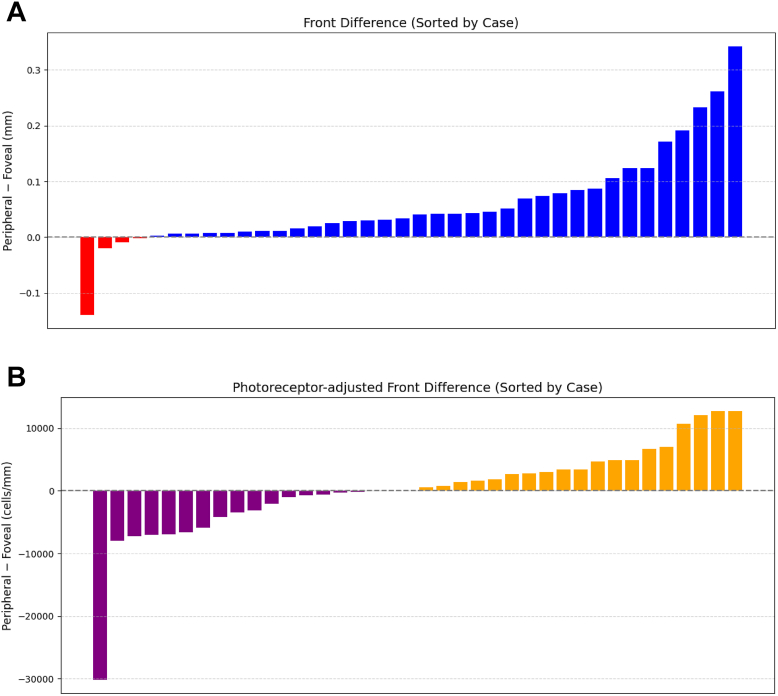
Difference plots comparing peripheral and foveal progression. **A,** Difference plots show per-case differences between peripheral and foveal directions (peripheral – foveal). Front exhibited predominantly positive differences, indicating peripheral progression. **B,** Whereas photoreceptor-adjusted front showed a more symmetric distribution around 0, indicating reduced directional bias.

In contrast, this directional difference was no longer observed when using the photoreceptor-adjusted front, with no significant difference between the foveal and peripheral directions (*P* = 0.537). This trend was further supported by individual difference plots (peripheral – foveal): while most cases showed positive values for the unadjusted front (indicating preferential peripheral progression), the photoreceptor-adjusted front exhibited a more symmetric distribution of differences, suggesting a reduction in directional bias (Fig 9). Moreover, when cosθ was treated as a continuous variable, the unadjusted front demonstrated a clear directional dependence, with progression distance gradually decreasing as cosθ increased. In contrast, the photoreceptor-adjusted front exhibited a markedly attenuated slope across the entire cosθ range, indicating a reduction in directional dependence (Fig S4, available at www.ophthalmologyscience.org).

### Representative Cases Demonstrating Differences between Front and Photoreceptor-Adjusted Front

**Figure 10:**
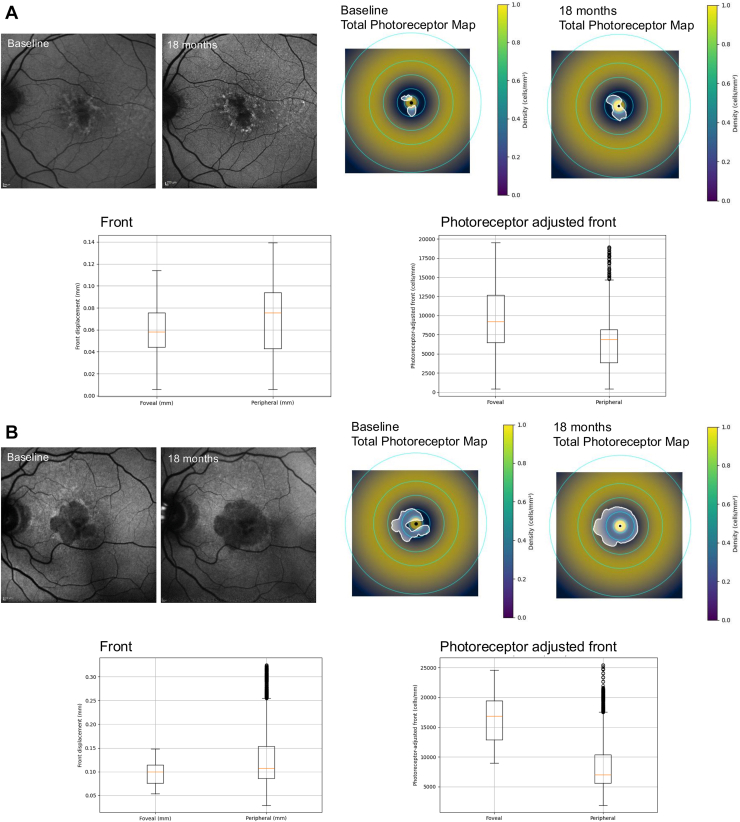
Representative cases in which the directionality of progression differed between front and photoreceptor-adjusted front. **A** and **B,** In these cases, the apparent direction of progression differed between the geometric front and the photoreceptor-adjusted front, resulting in a reversal of the dominant expansion pattern.

An additional noteworthy finding in this study was the presence of cases in which the directional trend observed with the unadjusted front was reversed when using the photoreceptor-adjusted front. A representative case illustrating this reversal is presented in Figure 10.

## Discussion

This study provides a photoreceptor density–based perspective on GA progression, suggesting that both the rate and directionality of lesion expansion may partly reflect the spatial distribution of photoreceptors. The results demonstrated a significant association between local photoreceptor density and the speed of GA progression, indicating that lesion expansion may be related to the regional distribution of photoreceptors within the retina. Moreover, the application of the photoreceptor-adjusted front metric reduced the previously reported asymmetry between foveal and peripheral progression, decreasing the directional variability of GA expansion. These findings raise the possibility that directional differences in GA progression are not solely attributable to geometric factors but may also be associated with underlying gradients of local photoreceptor density.

Previous studies have reported that the rate of GA expansion depends on lesion location, with extrafoveal lesions progressing more rapidly than those involving the foveal center.^13^ In addition, extrafoveal lesions have been shown to preferentially expand toward the periphery rather than toward the fovea.^12^ Several hypotheses have been proposed to explain the relative resistance of the foveal region to GA progression, including the presence of specialized vascular support surrounding the fovea that meets local metabolic and perfusion demands,^25^ differences in photoreceptor composition—where rods are more vulnerable to degeneration while cones exhibit relative resistance^26^—and the high concentration of macular pigment in the central fovea, which may protect against phototoxic damage.^27^ Collectively, these hypotheses have supported the widely accepted concept of biological “foveal protection” as a potential explanation for the directional asymmetry of GA expansion.

In parallel, several prior studies have examined the topographic pattern of GA progression across different retinal regions.^28^, ^29^, ^30^ Sayegh et al reported a slower decline of foveal sparing within the central 1 mm compared with the central 3 mm, suggesting relative preservation of the foveal core.^29^ Although their analysis was area-based and performed at different spatial scales, these observations appear generally consistent with our ring-wise findings, which suggest more pronounced changes across the parafoveal region (approximately 1–3 mm). Similarly, ETDRS grid–based analyses demonstrated faster GA progression in the parafoveal inner zone compared with the foveal center and more peripheral regions.^30^ In line with these reports, we observed relatively limited change in the foveal center and greater expansion in the parafoveal region. By modeling progression as a continuous function of eccentricity rather than relying solely on fixed subfields, our approach offers an alternative framework for characterizing the topographic pattern of GA progression.

Our weighting scheme is based on inhomogeneous photoreceptor density maps that vary strongly with eccentricity and incorporate the canonical foveal cone peak and perifoveal rod-dominant distribution described in human histologic topography studies.^19^, ^20^, ^21^ Although we used total photoreceptor density (rods + cones) as a parsimonious substrate term, rod–cone asymmetry may be mechanistically relevant because rods dominate outside the foveal center and are preferentially affected early in age-related macular degeneration, with scotopic dysfunction often exceeding photopic dysfunction despite preserved acuity.^31^ This raises the possibility that rod-weighted versus cone-weighted formulations could differentially capture spatial kinetics, particularly in foveal-sparing phenotypes.^32^

In addition, Müller cells exhibit regionally specialized architecture and molecular profiles in the fovea, and classic as well as recent studies report distinctive Müller cell organization and/or lower density/marker expression in the foveal region compared with more eccentric retina, supporting the plausibility that glial support gradients could modulate central resilience and boundary behavior.^33^, ^34^, ^35^ We therefore interpret photoreceptor-density weighting as an anatomy-informed explanatory component rather than a single mechanistic determinant, and future work should evaluate rod-specific and cone-specific weighting and integrate structural/vascular markers to better separate photoreceptor substrate effects from foveal specialization and glial/vascular support.

In contrast, the findings of this study suggest that the apparent attenuation of foveal progression may not be explained solely by proposed protective mechanisms but may also relate to the spatial distribution of photoreceptors surrounding the fovea, particularly the steep gradient of cone density. When photoreceptor density was not accounted for, GA appeared to progress more rapidly in the peripheral direction. However, after adjusting for local photoreceptor density, this directional asymmetry was attenuated, suggesting that part of the apparent difference in expansion may reflect the underlying density gradient. Taken together, these observations support the concept that the directionality of GA progression may be influenced by spatial inhomogeneity in retinal photoreceptor density. We refer to this framework as “density-driven kinetics,” while acknowledging that additional biological mechanisms may also contribute to regional differences in progression.

In the present cohort, modeled photoreceptor density explained approximately one-quarter of the variance in ring-wise growth, representing a moderate effect size. Published effect sizes for other imaging biomarkers of GA progression are variable and depend on whether analyses are global or spatially resolved, and on phenotype. OCT angiography-derived choriocapillaris flow deficit metrics have shown moderate-to-strong global associations with enlargement rate in selected cohorts (e.g., reported r values up to ∼0.75 in some regions), whereas spatially resolved local correlations along the lesion margin are typically modest (r ∼0.30–0.53) when spatial autocorrelation is accounted for.^36^^,^^37^ Outer retinal and ellipsoid zone-based biomarkers are similarly heterogeneous: baseline ellipsoid zone disruption area may correlate poorly with 1-year GA enlargement in unselected cohorts, yet correlations can be substantially stronger in phenotype-defined subsets (e.g., absence of reticular pseudo drusen). Quantitative outer retinal thickness metrics around GA have shown moderate associations with growth (e.g., r ˜ –0.46), and mixed-model analyses support predictive value of junctional-zone photoreceptor degeneration features.^38^, ^39^, ^40^ Histopathologic substrates such as basal laminar deposit are strongly implicated in age-related macular degeneration biology but are less standardized for routine trial enrichment compared with OCT angiography/OCT-derived surrogates.^39^^,^^41^ Collectively, these data suggest that photoreceptor-density weighting should be viewed as a complementary, anatomy-informed component that may add incremental value when combined with established vascular and outer-retinal biomarkers rather than a single dominant predictor. Because lesion morphology (e.g., perimeter) substantially influences apparent growth variability, multivariable enrichment models should test the incremental contribution of density-weighted features beyond morphology and established imaging biomarkers (e.g., ΔR^2^ or likelihood ratio tests).^42^

A substantial proportion of variability in GA progression remains unexplained. As a multifactorial disease, GA progression is likely influenced by additional mechanisms including vascular dysfunction, metabolic abnormalities, inflammatory processes, and RPE–Bruch's membrane–related alterations. Integration of these complementary factors may further enhance the explanatory and predictive performance of future GA progression models.

In contrast, the photoreceptor density used in this study represents an anatomic property of the retina that exists prior to these changes and may be associated with spatial variation in both the rate and directionality of GA expansion. Traditionally, structural assessment of GA progression has relied primarily on measures such as lesion area enlargement and foveal involvement. However, these structural indices do not always accurately reflect functional decline, and a dissociation between structural changes and visual function has been widely acknowledged.^11^ In fact, significant loss of best-corrected visual acuity is typically observed only after the lesion reaches the foveal center, whereas functional deficits such as scotomas and reduced retinal sensitivity often emerge earlier in the parafoveal region.^43^

To capture these early functional impairments, alternative metrics such as reading acuity, reading speed, and microperimetry-based sensitivity assessments have been emphasized as clinically meaningful endpoints.^43^^,^^44^ The photoreceptor density–based metric proposed in this study offers a complementary approach to conventional structural measures. Unlike traditional metrics that quantify only the geometric extent or direction of lesion expansion, this approach estimates the potential number of photoreceptors affected by GA progression.

Because photoreceptor distribution across the retina is highly nonuniform—particularly due to the steep increase in cone density near the fovea—the same 1 mm^2^ ΔArea may correspond to substantially different numbers of photoreceptors depending on lesion location. For example, expansion occurring near the fovea (within the 0.5–1.5 mm ring) may be associated with considerably greater photoreceptor loss compared with an equivalent lesion size in more peripheral regions (e.g., 4–5 mm ring). By incorporating this structural asymmetry into lesion analysis, the photoreceptor density–based metric may provide a biologically informed framework for interpreting GA progression and could serve as a useful adjunct for estimating the potential functional implications of lesion expansion.

Furthermore, this study observed cases in which the apparent pattern of lesion expansion differed when comparing the unadjusted front and the photoreceptor-adjusted front in the foveal and peripheral directions. Prior studies analyzing GA lesions that circumferentially surround the fovea have reported greater apparent expansion in the peripheral direction compared with the foveal direction.^12^ However, in representative cases from the present study, this directional difference was attenuated—or, in some instances, appeared altered—when analysis incorporated photoreceptor density–adjusted metrics. These observations suggest that, beyond measuring the geometric “distance” of lesion expansion, consideration of the regional photoreceptor density into which the lesion extends may provide additional context for interpreting progression patterns. This photoreceptor-informed framework may contribute to a more nuanced understanding of the potential functional implications of GA progression.

This study also introduces a quantitative metric based on photoreceptor density models of the human retina. This approach enables the estimation of photoreceptor impact that may not be adequately captured by conventional area-based assessments. For example, it may reflect the fact that even minor lesion expansion near the foveal center could be biologically and functionally more severe than an equivalent expansion in the peripheral retina.

Trial enrichment in GA commonly leverages baseline lesion characteristics (size, multifocality, location, FAF pattern, fellow-eye status) and often selects noncentral/fovealsparing lesions because effective-radius growth is reported to be ∼30% to 60% higher in noncentral GA and directional kinetics can favor faster peripheral spread.^39^^,^^42^ However, substantial interindividual variability persists even within enriched cohorts.^2^ We propose photoreceptor-density weighting as a complementary, low-cost stratification and modeling tool rather than a replacement for established approaches such as fixation-related metrics or choriocapillaris-based stratification.

Practically, a baseline density-exposure metric could be computed along the lesion margin (or within a perilesional band) and used for randomization stratification or as a prespecified covariate in mixed-effects models. Because lesion geometry and baseline perimeter materially influence apparent growth and motivated square-root transformation and alternative perimeter-adjusted metrics, density-weighted border-displacement endpoints may further reduce unexplained variance when used alongside established morphology covariates.^42^ Incremental utility should be quantified directly (e.g., ΔR^2^ or likelihood ratio testing) in models that already include standard baseline predictors and, where available, OCT angiography-derived choriocapillaris measures.

This study has several limitations that should be acknowledged. First, we did not directly evaluate the impact of GA progression on visual function, as the MAHALO dataset available at the reading center did not include functional outcome measures such as visual acuity or microperimetry. The present study should be regarded as a hypothesis-generating and model-development investigation. Accordingly, evaluation of predictive performance and assessment of clinical utility, including associations with visual function, remain important topics for future research. Therefore, validation using an independent cohort that includes visual function data will be necessary.

Second, photoreceptor density distributions are known to vary among individuals, influenced by factors such as age, sex, and axial length.^19^^,^^45^^,^^46^ In this study, we used a theoretical average distribution model for photoreceptor density, and therefore, the findings may not be fully generalizable to all individual cases. Specifically, incorporation of individualized anatomical parameters—such as those obtained through adaptive optics–based photoreceptor imaging—together with validation in independent external cohorts, may enable construction of more precise and patient-specific models of GA progression that better reflect true photoreceptor distributions in elderly patients with GA. Furthermore, the theoretical density maps used in this study were derived from younger donor eyes, and age-related or disease-related alterations in photoreceptor distribution were not directly accounted.^19^, ^20^, ^21^ Because the present analysis was limited to fellow eyes from the MAHALO trial, which may represent a specific phenotype, validation in cohorts in clinical settings is essential to confirm generalizability. Future investigations integrating vascular dysfunction, metabolic alterations, inflammatory pathways, and RPE–Bruch's membrane microstructural changes will be necessary to further refine disease modeling. Furthermore, given the role of photoreceptor loss in GA expansion, future studies should include the development of complementary RPE-based models and comparative analyses incorporating rod-only and cone-only density–based approaches.

Third, in the directional subanalysis comparing foveal and peripheral progression, we employed a classification approach based on cosθ to define directionality. While this method entails a degree of simplification, previous studies have also emphasized the necessity of explicitly defining direction to enable valid comparisons between foveal and peripheral expansion.^12^ Our methodology followed a similar framework. However, when direction is treated as a continuous variable, direct binary comparisons between foveal and peripheral directions become methodologically problematic. To address this, we performed a supplementary analysis in which cosθ was treated as a continuous variable at the pixel level, and its relationship with both front and photoreceptor-adjusted front was evaluated. This analysis served to reinforce the validity of the direction-based classification results.

Finally, while a prior study conducted directional analysis in 46 eyes,^12^ our directional comparison was limited to 38 eyes with extra-foveal GA. Therefore, we cannot completely exclude the possibility that interindividual variability influenced the results. The present findings should be regarded as exploratory observations suggesting an association between GA expansion directionality and photoreceptor density. Further validation in larger cohorts will be necessary to confirm the robustness and generalizability of these associations.

In conclusion, this study introduced a photoreceptor density-based metric for evaluating GA progression and identified spatial patterns of photoreceptor involvement that may not be fully captured by conventional measures. Our findings suggest that local photoreceptor density gradients may represent one of several factors contributing to GA expansion, providing a biologically informed framework for interpreting structural progression and its potential functional implications.

## Supplementary Data

Supplementary Table S3

Figure S1A Visualization of ECC registration accuracy across time points. Although the primary analysis was performed using baseline and 18-month images, image registrations across all available time points for the same eye (baseline, 6, 12, and 18 months) are presented to demonstrate the validity of the registration accuracy. (A) Upper panels show fundus autofluorescence (FAF) images at baseline, 6, 12, and 18 months, demonstrating apparent lesion misalignment across time points, with corresponding binary masks shown below. Without image registration, residual misalignment hampers accurate assessment of lesion expansion, as reflected in the progression heatmap.

Figure S1B Visualization of ECC registration accuracy across time points. Although the primary analysis was performed using baseline and 18-month images, image registrations across all available time points for the same eye (baseline, 6, 12, and 18 months) are presented to demonstrate the validity of the registration accuracy. (B) After registration using the enhanced correlation coefficient (ECC) algorithm, lesion locations were spatially aligned across all visits, enabling accurate visualization and assessment of lesion expansion on the resulting progression heatmaps.

Figure S3 Overview of cosine-based vector direction analysis. (A) The direction of GA lesion expansion was classified using the cosine of the angle (θ) between 2 vectors. Vector A represents the vector from the foveal center to each front pixel, and vector B represents the local direction of lesion expansion. (B) Directional classification was based on the cosine value (cosθ) calculated from these vectors. (C) Acute angles (0°–90°) indicating macular-directed expansion and obtuse angles (90°–180°) indicating peripheral-directed expansion. This vector-based analysis was applied to all pixels along the GA lesion front. (D) Representative examples illustrate the directional classification, with blue indicating macular direction and orange indicating peripheral direction.

Figure S4 Supplementary analysis of GA expansion direction using cosθ as a continuous variable. PR-adjusted = photoreceptor-adjusted front. When cosine values were analyzed as a continuous variable, front showed a clear directional dependence, with progression decreasing as cosθ increased, whereas this slope was attenuated across the entire cosθ range for the photoreceptor-adjusted front.
