# Supplementary material for: Photoreceptor Density–Dependent Kinetics of Geographic Atrophy Progression
Source: Ophthalmol Sci. 2026 Apr 17;6(7):101198. doi: 10.1016/j.xops.2026.101198 (PMC13234230; doi:10.1016/j.xops.2026.101198)
Supplement: Supplementary Table S1 [file mmc1.pdf]

Supplementary Table 1. Summary of ECC-Based Registration Accuracy

Timepoint	ECC correlation	ECC error (Mean ± SD, Max)
**6M**	0.989	2.29 ± 1.93 px, Max = 10.0
**12M**	0.970	5.13 ± 3.79 px, Max = 20.2
**18M**	0.972	4.96 ± 3.34 px, Max = 21.0

The Enhanced Correlation Coefficient (ECC) correlation coefficient indicates the accuracy of structural alignment between baseline and follow-up images, with a value of 1.0 representing perfect registration. Values greater than 0.97 are generally considered to reflect high-precision registration. In the present study, the primary analysis was performed using baseline and 18-month images; however, to demonstrate the validity of the registration accuracy, ECC correlation coefficients across all available time points for the same eyes (baseline, 6, 12, and 18 months) are presented. High ECC correlation values were consistently observed at all time points, supporting the reliability of high-precision image registration using the ECC algorithm.
