# Supplementary material for: Photoreceptor Density–Dependent Kinetics of Geographic Atrophy Progression
Source: Ophthalmol Sci. 2026 Apr 17;6(7):101198. doi: 10.1016/j.xops.2026.101198 (PMC13234230; doi:10.1016/j.xops.2026.101198)
Supplement: Supplementary Table S2 [file mmc2.pdf]

Supplementary Table2. Photoreceptor Density Distribution

Distance from Foveal Center (mm)	Cone Density (cones/mm²)	Rod Density (rods/mm²)	Total Density (cells/mm²)
**0**	199,000	0	199,000
**0.5**	70,000	50,000	120,000
**1.0**	20,000	90,000	110,000
**2.0**	10,000	140,000	150,000
**3.0**	8,000	160,000	168,000
**5.0**	6,000	150,000	156,000

Photoreceptor densities are based on established histological models and are provided as reference values for spatial weighting of lesion expansion analyses.
