# Supplementary material for: Photoreceptor Density–Dependent Kinetics of Geographic Atrophy Progression
Source: Ophthalmol Sci. 2026 Apr 17;6(7):101198. doi: 10.1016/j.xops.2026.101198 (PMC13234230; doi:10.1016/j.xops.2026.101198)
Supplement: Supplementary Table S3 [file mmc3.pdf]

Supplementary Table3. Interpretation of Cosine Values for Directional Classification

Angle (θ)	cos θ Value	Vector Relationship	Interpretation
**0°–90° (Acute)**	$\cos\theta > 0$	Vectors point in similar direction	Foveal directed
**90° (Right)**	$\cos\theta = 0$	Vectors are orthogonal	Neutral direction
**90°–180° (Obtuse)**	$\cos\theta < 0$	Vectors point in opposite direction	Peripheral-directed

Directional classification is based on the cosine of the angle (θ) between the vector from the foveal center to each lesion front pixel and the local expansion vector.
