# Supplementary figures and images for: Photoreceptor Density–Dependent Kinetics of Geographic Atrophy Progression
Source: Ophthalmol Sci. 2026 Apr 17;6(7):101198. doi: 10.1016/j.xops.2026.101198 (PMC13234230; doi:10.1016/j.xops.2026.101198)

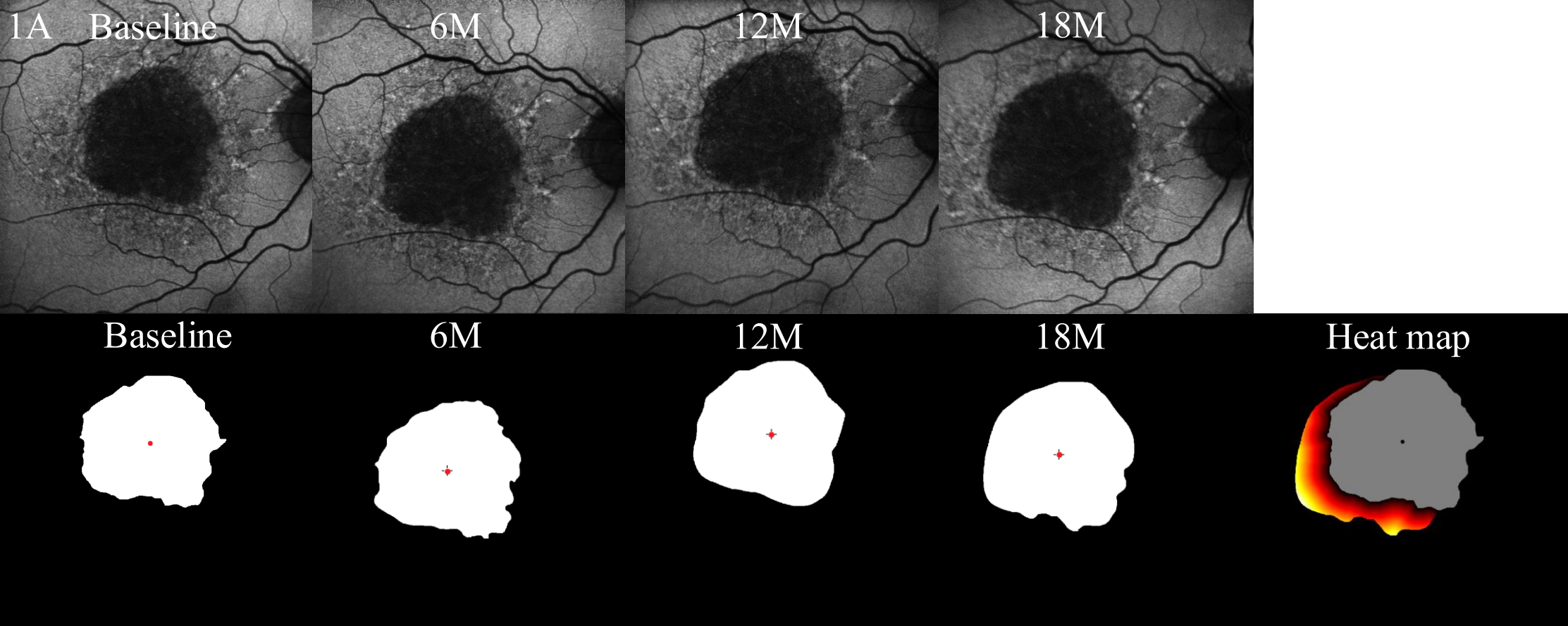

The mask images were not registered, resulting in spatial misalignment.

Supplement: Figure S1A — Visualization of ECC registration accuracy across time points. Although the primary analysis was performed using baseline and 18-month images, image registrations across all available time points for the same eye (baseline, 6, 12, and 18 months) are presented to demonstrate the validity of the registration accuracy. (A) Upper panels show fundus autofluorescence (FAF) images at baseline, 6, 12, and 18 months, demonstrating apparent lesion misalignment across time points, with corresponding binary masks shown below. Without image registration, residual misalignment hampers accurate assessment of lesion expansion, as reflected in the progression heatmap. [file mmc4.pdf]

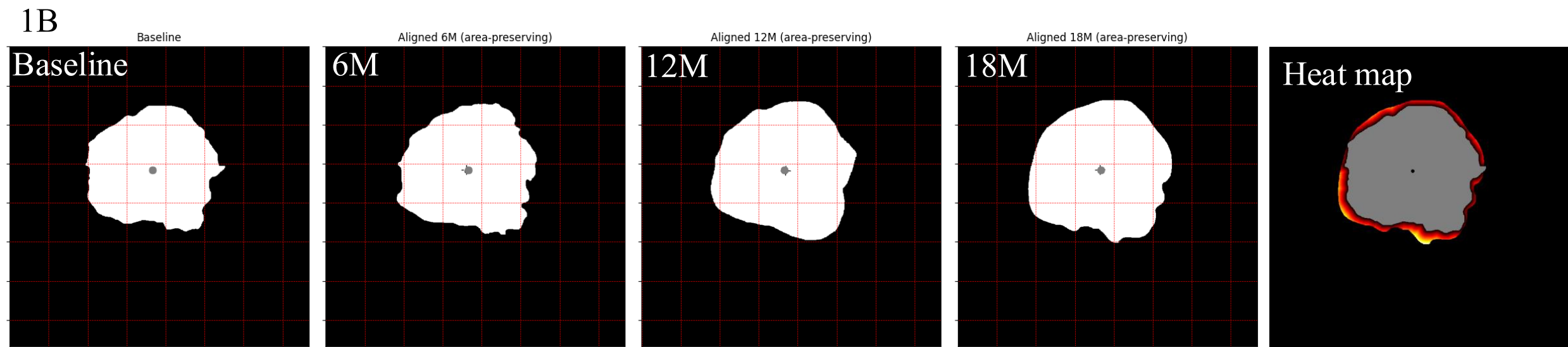

All mask images were aligned using Enhanced Correlation Coefficient algorithm.

Supplement: Figure S1B — Visualization of ECC registration accuracy across time points. Although the primary analysis was performed using baseline and 18-month images, image registrations across all available time points for the same eye (baseline, 6, 12, and 18 months) are presented to demonstrate the validity of the registration accuracy. (B) After registration using the enhanced correlation coefficient (ECC) algorithm, lesion locations were spatially aligned across all visits, enabling accurate visualization and assessment of lesion expansion on the resulting progression heatmaps. [file mmc5.pdf]

Directional dependence is attenuated after PR adjustment

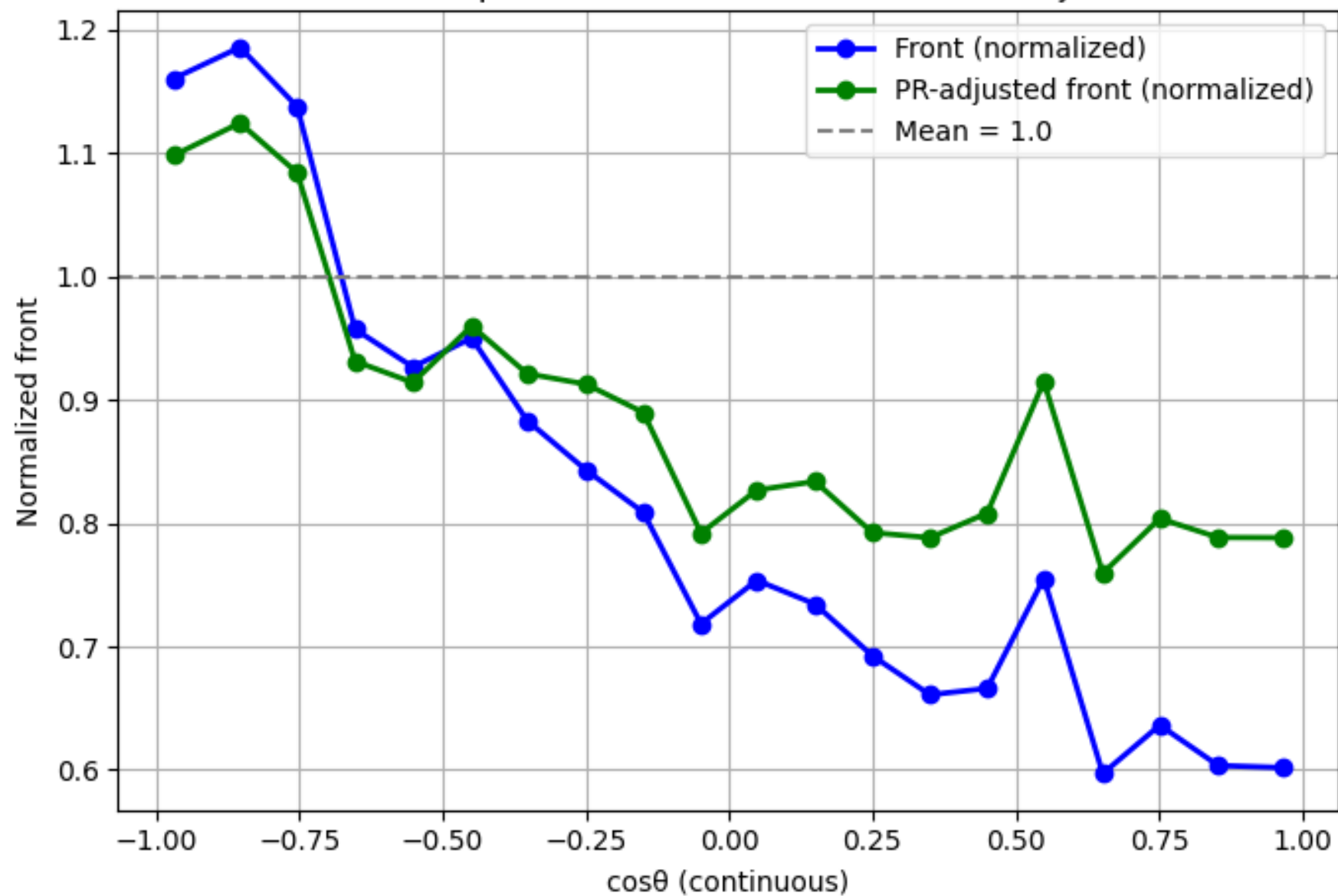

Supplement: Figure S4 — Supplementary analysis of GA expansion direction using cosθ as a continuous variable. PR-adjusted = photoreceptor-adjusted front. When cosine values were analyzed as a continuous variable, front showed a clear directional dependence, with progression decreasing as cosθ increased, whereas this slope was attenuated across the entire cosθ range for the photoreceptor-adjusted front. [file mmc7.pdf]
