# Supplementary material for: Photoreceptor Density–Dependent Kinetics of Geographic Atrophy Progression
Source: Ophthalmol Sci. 2026 Apr 17;6(7):101198. doi: 10.1016/j.xops.2026.101198 (PMC13234230; doi:10.1016/j.xops.2026.101198)

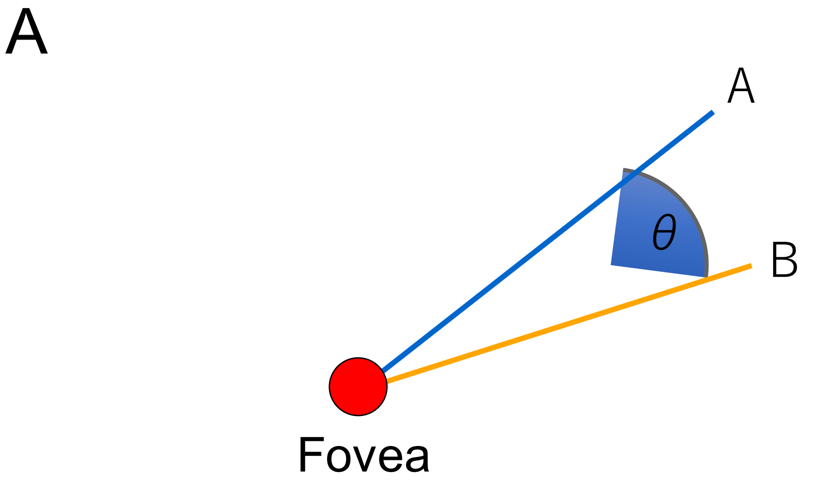

cosθ = (Vector A • Vector B) / (|A| × |B|)

θ is the angle between:

- Vector A: from fovea to each front pixel
- Vector B: local front expansion direction

→ The cosine of this angle determines directional classification.

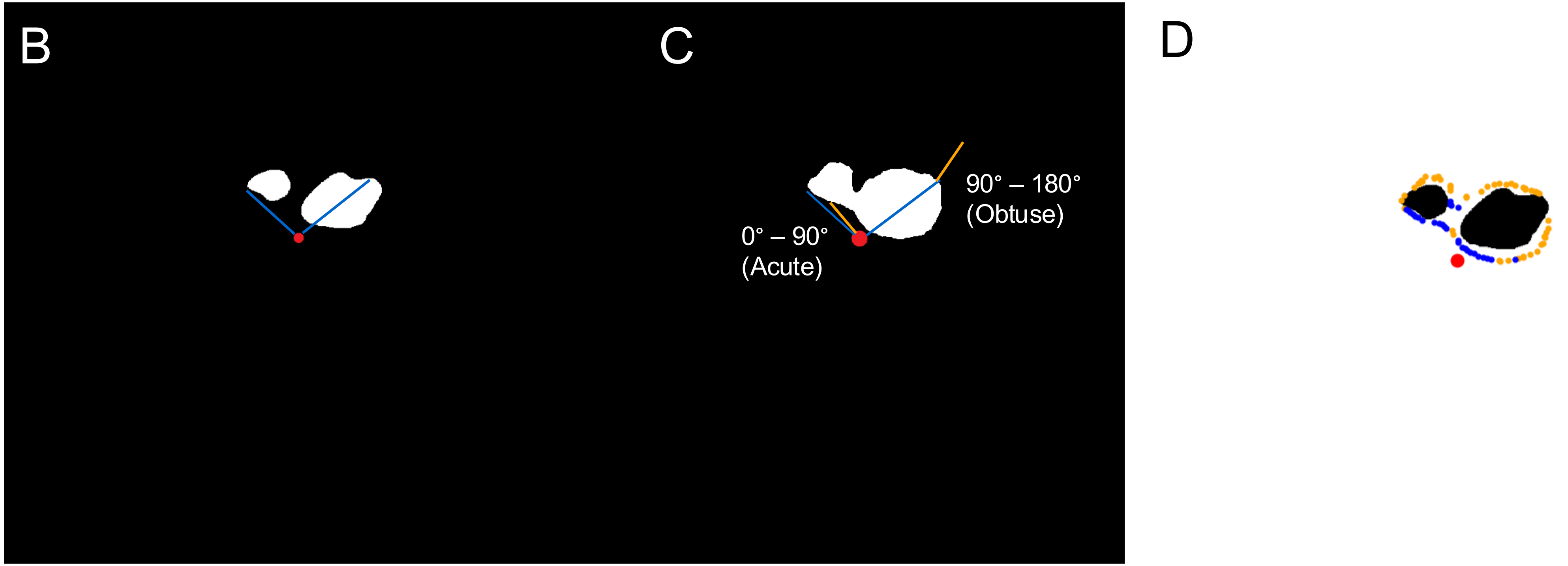

Supplement: Figure S3 — Overview of cosine-based vector direction analysis. (A) The direction of GA lesion expansion was classified using the cosine of the angle (θ) between 2 vectors. Vector A represents the vector from the foveal center to each front pixel, and vector B represents the local direction of lesion expansion. (B) Directional classification was based on the cosine value (cosθ) calculated from these vectors. (C) Acute angles (0°–90°) indicating macular-directed expansion and obtuse angles (90°–180°) indicating peripheral-directed expansion. This vector-based analysis was applied to all pixels along the GA lesion front. (D) Representative examples illustrate the directional classification, with blue indicating macular direction and orange indicating peripheral direction. [file mmc6.pdf]
